# Extrapulmonary Manifestations of *Mycoplasma pneumoniae* : A 3-Year-Old Male Presenting with Mycoplasma Pneumonia, Urticaria Multiforme, and Bicytopenia

**DOI:** 10.1055/s-0046-1822659

**Published:** 2026-06-03

**Authors:** Salsabeel Sweidan, Menatalla Dyab, Anupama Dwarki, Yaseen Rafee

**Affiliations:** 1 Department of Pediatrics, Hurley Children's Hospital, College of Human Medicine, Michigan State University, Flint, Michigan, United States

**Keywords:** mycoplasma, cytopenia, extrapulmonary manifestations, *Mycoplasma pneumoniae*, urticaria multiforme

## Abstract

In our case report, we highlight how mycoplasma presents as a spectrum, ranging from the most benign to the most severe presentations. We report a rare case of a 3-year-old boy with *Mycoplasma pneumoniae* infection who presented with urticaria multiforme and bicytopenia. It also points out the wide range of possible clinical manifestations of *M. pneumoniae* in pediatric patients. The report also summarizes a review of the only two published pediatric cases that presented with pancytopenia in the English literature.

## Introduction

*Mycoplasma pneumoniae* is a well-recognized pathogen that colonizes the mucosal surfaces of both humans and animals. It infects the upper and lower respiratory tracts of children and adults, resulting in a wide range of respiratory and non-respiratory clinical conditions. These extrapulmonary symptoms include hematologic, neurologic, gastrointestinal, musculoskeletal, dermatologic, and cardiac manifestations. While *M. pneumoniae* infection is frequently considered in the differential diagnoses of patients with respiratory illnesses and commonly managed empirically with macrolides and fluoroquinolones, it is rarely suspected in patients presenting with non-respiratory symptoms. 1

In this report, we describe a case of a 3-year-old male who presented with *M. pneumoniae* respiratory symptoms, urticaria multiforme, and pancytopenia. We aim to highlight the variability in presentation and emphasize the importance of considering this diagnosis as part of the differential in similar pediatric cases.

## Case Presentation

A 3-year-old male patient was admitted to the hospital with symptoms that began 1 week before presentation. His initial complaint was lethargy, which progressed to reduced oral intake, abdominal pain, vomiting, and a progressive generalized itchy rash.

**Fig. 1:**
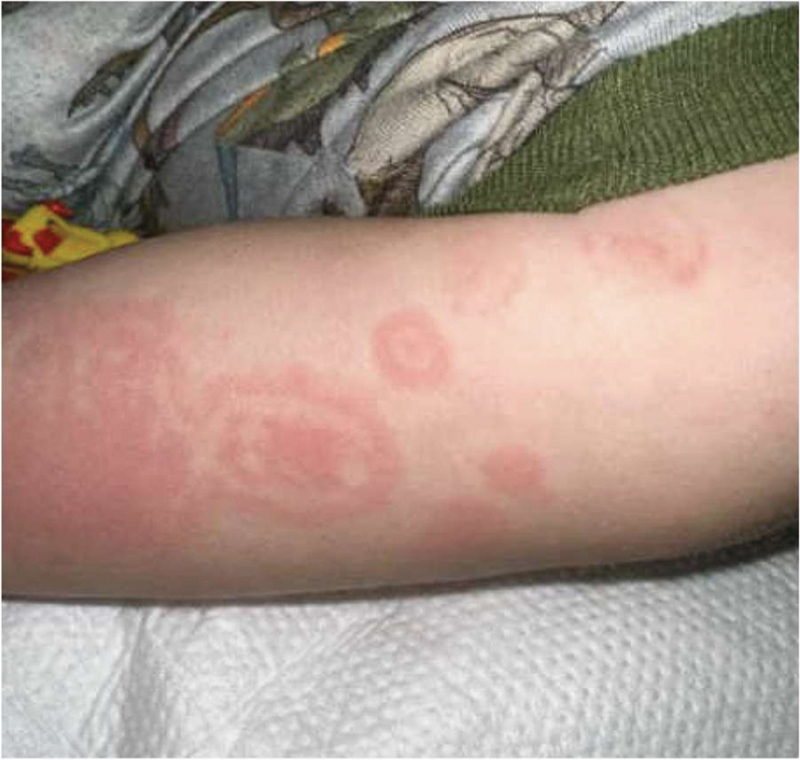
Generalized, migratory, erythematous rash with target-like lesions.

On examination, the patient appeared lethargic with a temperature of 38°C, heart rate of 157 beats per minute, and respiratory rate of 20 per minute. He had a generalized, migratory, erythematous rash with target-like lesions ( Fig. 1 ), cervical lymphadenopathy, and hepatomegaly. Abdominal examination revealed generalized tenderness without guarding or rebound tenderness. Respiratory examination noted bilaterally decreased air entry at the lung bases. Additionally, easy bruising was observed, including purpuric lesions at the site of blood pressure cuff.

**Fig. 2:**
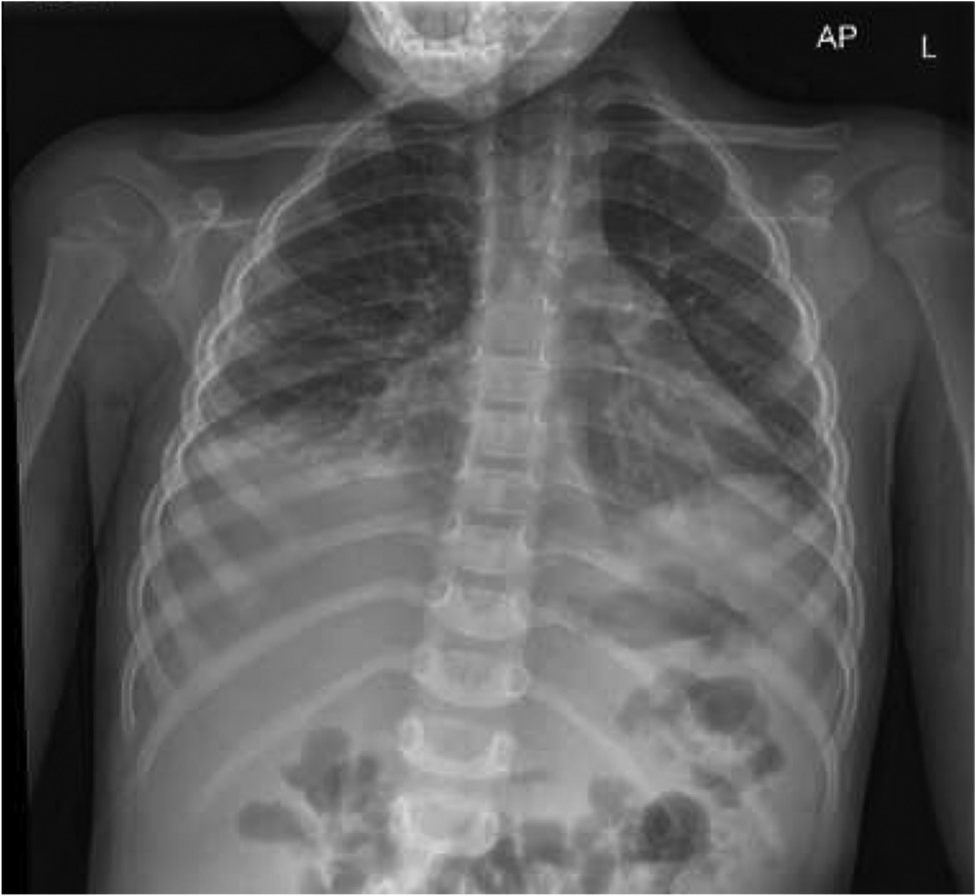
Small-to-moderate bilateral pleural effusions with bilateral perihilar peribronchial cuffing and patchy infiltrates in the lower lung zones.

Laboratory investigations showed leukopenia 3.7 K/µL (6.0–18.0 K/µL) associated with neutropenia 0.5 K/µL (1.8–8.0 K/µL), lymphocytopenia 2.4 K/µL (3.2–9.5 K/µL), and thrombocytopenia 54 K/µL (130–430 K/µL). Hemoglobin (HB) was measured at 11.9 g/dL (10.8–14.8 g/dL). He also had transaminitis, with an ALT of 84 µ/L (7–40 µ/L) and AST 108 µ/L (0–40 µ/L). CRP was elevated at 53.08 mg/L (0–10.0 mg/L), and lactate dehydrogenase (LDH) was 340 µ/L (100–225 µ/L). Imaging studies, including computed tomography (CT) of the abdomen and pelvis with contrast, showed moderate abdominal ascites, and chest ultrasound and X-ray revealed small-to-moderate bilateral pleural effusions with bilateral perihilar peribronchial cuffing and patchy infiltrates in the lower lung zones ( Fig. 2 ).

Upon admission, the patient was provisionally treated with intravenous (IV) azithromycin, and IV cefepime was added a day later due to persistent fever and neutropenia. Furthermore, he received symptomatic management for the itchy urticaria multiforme rash with cetirizine, famotidine, and hydroxyzine. A hematology consultation was obtained for pancytopenia and elevated LDH. The findings were attributed to an underlying infectious or autoimmune etiology for the systemic inflammatory response, rather than a primary hematological disorder. Expanded microbial studies, including respiratory viral panel, blood culture, Epstein–Barr virus antibodies, parvovirus B19 antibodies, Legionella antigen in urine, and mononucleosis screening, were done and were negative. Finally, *M. pneumoniae* IgG and IgM were positive.

Serial CBCs showed improving neutropenia; therefore, cefepime was switched to ceftriaxone, and azithromycin was continued for the treatment of community-acquired pneumonia in the setting of *M. pneumoniae* infection.

Throughout his admission, he demonstrated gradual clinical and laboratory improvement. The patient was later discharged to continue azithromycin and amoxicillin. A complete blood count, performed 1 week after discharge, showed full recovery of white blood cells (WBCs) and platelet counts.

## Discussion

*M. pneumoniae* is associated with diverse extrapulmonary manifestations, despite pneumonia being the hallmark of the infection. Central nervous system involvement, for example, occurs in up to 7% of hospitalized patients with *M. pneumoniae* infection and may manifest as encephalitis, transverse myelitis, and cerebellar ataxia. Cardiac manifestations are less common (<10%), but may include myocarditis, pericarditis, complete heart block, and hemopericardium. 2 Musculoskeletal manifestations have also been reported, such as arthritis and rhabdomyolysis. 3 Immune hemolytic anemia is an uncommon but recognized hematological complication, often mediated by cold agglutinin. 3 Several cases of *Mycoplasma* -associated hemolytic anemia and hemophagocytic lymphohistiocytosis (HLH) have been reported, but only two cases of *Mycoplasma* -associated pancytopenia have been documented in a pediatric patient upon our review of the English literature. 4 5 The first reported case was in 2009 in a previously healthy 9-year-old girl who presented to the emergency department with a 1-week history of dyspnea, cough, and fever. The chest X-ray showed areas of infiltration (lobar pneumonia). A blood count at diagnosis showed a WBC count of 1.1 K/µL, an absolute neutrophil count of 0.4 K/µL, and a platelet count of 68 K/µL. The HgB level was 9.1 g/dL. The patient was diagnosed with pneumonia and pancytopenia due to cold agglutinin and mycoplasma infection based on positive serologies. She needed a blood transfusion and intravenous immunoglobulin (1 g/kg) on day 3 of the admission (HB was 5.5 g/dL). She continued 2 weeks of antibiotics, and on the day of discharge, she showed complete recovery of all blood cell counts. 4 The second reported case was in 2017, when a 12-year-old boy was admitted to the general pediatrics inpatient unit for fever of unknown origin and pancytopenia. He also developed bilateral, non-exudative conjunctivitis, most pronounced in the limbus. However, he did not have any other clinical signs suggestive of Kawasaki disease. Admission laboratory results were significant for microcytic anemia with reticulocytopenia, lymphocytopenia, and thrombocytopenia on the complete blood count, along with elevated inflammatory markers. A thorough infectious workup was negative; rheumatologic laboratories were also unrevealing. Peripheral blood smear did not demonstrate any blast cells, and flow cytometry immunophenotyping did not identify abnormal cell populations. Hemoglobin electrophoresis was remarkable for hemoglobin C trait with possible coexisting α thalassemia explaining the microcytosis noted on the complete blood count. By the time of discharge, the patient's platelet count had recovered, and his WBC count remained stable, with resolution of lymphocytopenia. He remained mildly anemic, likely due to his underlying hemoglobinopathy, though he began to show evidence of reticulocytosis by day 5 to 6. After discharge, the patient's *mycoplasma* IgM and IgG returned positive. He completed a 5-day course of azithromycin and was followed by both his primary care physician and the hematology team. 5

Mucocutaneous involvement is yet another common presentation. In 2015, a comprehensive literature review identified 202 cases across 95 reports that proposed the term “Mycoplasma-Induced Rash and Mucositis” (MIRM) to differentiate these eruptions from drug-induced “Stevens-Johnson syndrome” or “erythema multiforme” 6 7 . In pediatric patients, *M. pneumoniae* has also been associated with urticaria multiforme, a benign cutaneous hypersensitivity reaction that is characterized by the acute and transient onset of blanchable, annular, polycyclic, erythematous wheals with dusky, ecchymotic centers in association with acral edema that is frequently misdiagnosed as erythema multiforme. 8

There are three known mechanisms that contribute to the development of extrapulmonary manifestations of mycoplasma:

- *Direct effect* —presence of the bacteria at the site of inflammation that triggers the immune and inflammatory response.
- *Indirect effect* —it is caused by an immune system reaction and immune complex formation to bacteria that are not present at the site of inflammation.
- *Vascular occlusion* —The resultant infection and inflammation cause blood flow blockage and impairment either directly or through immune-mediated mechanisms. 9

Since respiratory symptoms may be mild or absent in extrapulmonary *M. pneumoniae* infections, serological tests are preferred for diagnosis.

Literature also suggests that in severe cases such as encephalitis, treatment may include both antibiotics and immunomodulators (e.g., corticosteroids or immunoglobulins). 9 10

Our case is unique in the fact that it represents a rare range of manifestations of extrapulmonary findings that involve multiple organs in the same patient. It involved cutaneous, liver, and hematological manifestations in addition to the pulmonary findings, which were not reported before. Our patient recovered well, and upon 6 months' phone follow-up, the patient's mother reported that he is in good health with no further concerns.

## Conclusion

Mycoplasma's primary presentation, as seen in the majority of cases, is pneumonia; however, there have been some cases in which it has presented with other extrapulmonary manifestations, which may not always be correctly identified by physicians, resulting in unnecessary further investigations, delayed diagnosis, and potential delays in appropriate treatment. Our case represents a rare occurrence of extrapulmonary symptoms, including cutaneous manifestations such as urticarial rash and cytopenic presentation. Urticaria multiforme has long been recognized as a benign rash that is frequently mistaken for other dermatoses, such as erythema multiforme. The concept of “mistaken identity” underscores that mycoplasma infection can manifest across a spectrum, ranging from benign to severe presentations. It not only represents variability, but it also shows that we as physicians should consider this as part of a multitude of differential diagnoses when seen in these types of conditions with pediatric patients. Cases such as the one presented here highlight to physicians that further investigations need to be considered, and atypical presentations for infections, with the variability commonly seen in an acute setting, must be evaluated efficiently to ensure appropriate and timely patient care.

## References

[1] Sánchez-Vargas F M Gómez-Duarte O G Mycoplasma pneumoniae-an emerging extra-pulmonary pathogen Clin Microbiol Infect 2008 14 02 105 117 17949442 10.1111/j.1469-0691.2007.01834.x

[2] Krafft C Christy C Mycoplasma pneumonia in children and adolescents Pediatr Rev 2020 41 01 12 19 31894069 10.1542/pir.2018-0016

[3] Kashyap S Sarkar M Mycoplasma pneumonia: clinical features and management Lung India 2010 27 02 75 85 20616940 10.4103/0970-2113.63611 PMC2893430

[4] Gursel O Altun D Atay A A Bedir O Kurekci A E Mycoplasma pneumoniae infection associated with pancytopenia: a case report J Pediatr Hematol Oncol 2009 31 10 760 762 19755923 10.1097/MPH.0b013e3181b7eb4b

[5] Speir E Charvat C Varghese S Pancytopenia and fever of unknown origin in a 12-year-old boy Clin Pediatr (Phila) 2018 57 05 607 610 29073781 10.1177/0009922817738345

[6] Canavan T N Mathes E F Frieden I Shinkai K Mycoplasma pneumoniae-induced rash and mucositis as a syndrome distinct from Stevens-Johnson syndrome and erythema multiforme: a systematic review J Am Acad Dermatol 2015 72 02 239 245 25592340 10.1016/j.jaad.2014.06.026

[7] Santos R P Silva M Vieira A P Brito C Mycoplasma pneumoniae-induced rash and mucositis: a recently described entity BMJ Case Rep 2017 2017 bcr-2017-220768 10.1136/bcr-2017-220768 PMC5623246 28830900

[8] Emer J J Bernardo S G Kovalerchik O Ahmad M Urticaria multiforme J Clin Aesthet Dermatol 2013 6 03 34 39 23556035 PMC3613272

[9] Narita M Classification of extrapulmonary manifestations due to Mycoplasma pneumoniae infection on the basis of possible pathogenesis Front Microbiol 2016 7 23 26858701 10.3389/fmicb.2016.00023 PMC4729911

[10] Daba M Kang P B Sladky J Bidari S S Lawrence R M Ghosh S Intravenous immunoglobulin as a therapeutic option for Mycoplasma pneumoniae encephalitis J Child Neurol 2019 34 11 687 691 31185782 10.1177/0883073819854854 PMC9889999

